# Functional features of cancer stem cells in melanoma cell lines

**DOI:** 10.1186/1475-2867-13-78

**Published:** 2013-08-06

**Authors:** Rüdiger M Zimmerer, Philippe Korn, Philippe Demougin, Andreas Kampmann, Horst Kokemüller, André M Eckardt, Nils-Claudius Gellrich, Frank Tavassol

**Affiliations:** 1 Department of Oral and Maxillofacial Surgery, Head & Neck Oncology Laboratory, Hannover Medical School, Carl-Neuberg-Str. 1, Hannover D-30625, Germany; 2 Division of Molecular Psychology, Life Sciences Training Facility (LSTF), Missionsstrasse 60/62, Basel CH-4055, Switzerland

**Keywords:** Cancer stem cells, CD117, CD133, CD146, CD271, Cell lines, D10, Melanoma, WM115

## Abstract

**Background:**

Recent evidence suggests a subset of cells within a tumor with "stem-like" characteristics. These cells are able to transplant tumors in immunodeficient hosts. Distinct from non-malignant stem cells, cancer stem cells (CSC) show low proliferative rates, high self-renewing capacity, propensity to differentiate into actively proliferating tumor cells, and resistance to chemotherapy or radiation. They are often characterized by elevated expression of stem cell surface markers, in particular CD133, and sets of differentially expressed stem cell-associated genes. CSC are usually rare in clinical specimens and hardly amenable to functional studies and gene expression profiling. In this study, a panel of heterogenous melanoma cell lines was screened for typical CSC features.

**Methods:**

Nine heterogeneous metastatic melanoma cell lines including D10 and WM115 were studied. Cell lines were phenotyped using flow cytometry and clonogenic assays were performed by limiting dilution analysis on magnetically sorted cells. Spheroidal growth was investigated in pretreated flasks. Gene expression profiles were assessed by using real-time rt-PCR and DNA microarrays. Magnetically sorted tumor cells were subcutaneously injected into the flanks of immunodeficient mice. Comparative immunohistochemistry was performed on xenografts and primary human melanoma sections.

**Results:**

D10 cells expressed CD133 with a significantly higher clonogenic capacity as compared to CD133- cells. Na8, D10, and HBL cells formed spheroids on poly-HEMA-coated flasks. D10, Me39, RE, and WM115 cells expressed at least 2 of the 3 regulatory core transcription factors SOX2, NANOG, and OCT4 involved in the maintenance of stemness in mesenchymal stem cells. Gene expression profiling on CD133+ and CD133- D10 cells revealed 68 up- and 47 downregulated genes (+/-1.3 fold). Two genes, MGP and PROM1 (CD133), were outstandingly upregulated. CD133+ D10 cells formed tumors in NSG mice contrary to CD133- cells and CD133 expression was detected in xenografts and primary human melanoma sections using immunohistochemistry.

**Conclusions:**

Established melanoma cell lines exhibit, to variable extents, the typical features of CSCs. The tumorigenic cell line D10, expressing CD133 and growing in spheroids and might qualify as a potential model of melanoma CSCs.

## Background

Malignant melanoma occurs in 5% of men and 4% of women in the Western world [1]. Patients with advanced disease have a poor prognosis with a reported median survival ranging between 3 and 11 months [2]. In stage IV of the disease, when patients suffer from inoperable recurrent tumors and regional and distant metastases, therapeutic strategies include systemic chemotherapy and chemoimmunotherapy. Interferon-alpha and immune modulators such as interleukin 2 (IL-2) and anti-CTLA-4 (cytotoxic T-lymphocyte antigen-4) have shown a significant clinical benefit to the patients in some prospective randomized studies [3-6]. Although recent clinical trials using targeted therapy (vemurafenib) suggest promising results, the prognosis of patients with advanced disease remains poor [7]. In this context, the idea that tumors comprise cells endowed with stem cell-like properties, which might qualify as potential targets, is part of ongoing research. Hamburger and his group described, in 1977, that tumors comprise cells with heterogeneous tumorigenicity and differentiation potential [8]. By applying the principles of stem cell biology to cancer, many tumors have recently been shown to be organized hierarchically into clonally derived populations of cells with different tumorigenic potentials. Bonnet and colleagues [9] were the first who could phenotypically distinguish cells of acute myeloid leukemia (AML) with high tumorigenicity from the remaining tumor cells using surface markers. It was shown that only a small subset of these cells, phenotypically similar to hematopoietic stem cells, could transfer acute AML when transplanted into immunodeficient mice. It was suggested that the tumorigenic cell population represented a minority of cells within the tumor and that its isolation could be attempted from most tumors based on a unique surface marker expression pattern. In particular CD133 (Prominin 1), which is expressed on stem and early progenitor cells (CD34+ hematopoietic stem cells) and tumor-initiating cells of several malignancies [10,11], is prominent subject of ongoing research. A few more properties of CSCs have been identified so far, including their common capacity to grow in anti-adhesive structures called spheroids and a higher resistance to hypoxia, possibly related to aberrant angiogenesis in rapidly expanding tumors [12,13]. Fang [14] described a subset of cells derived from freshly isolated or in vitro stabilized melanoma cell lines that was able to form “melanoma spheroids” when grown in a specific stem cell medium. Tavaluc [15] and Zhou [16] suggest that CSCs have a higher ability to survive under hypoxic conditions than normal cancer cells.

Taken together, CSCs are defined by their ability to induce tumor growth following transplantation. The tumorigenic potential of CSCs unites self-renewal and differentiation potential. Although some tumorigenic phenotypes have been identified in several solid malignancies so far, CSCs cannot be clearly defined by a certain morphology, genotype, or phenotype [17]. Current cancer therapeutics based on tumor regression may target and kill differentiated tumor cells, which compose the bulk of the tumor, while sparing the rare CSC population. The CSC model suggests that the design of new cancer therapeutics may require the targeting and elimination of CSCs. The aim of the study was to identify CSC markers potentially allowing the functional characterization of specific cell subsets from clinical specimens, which have been identified in different types of tumors, including melanoma [18]. However, the minute numbers of cells presenting these features that can be obtained from surgical samples usually prevent a thorough evaluation of the molecular pathways involved in “stemness”. This background has prompted several research groups to explore the possibility of taking advantage of the relative heterogeneity of established cell lines to identify cell subsets endowed with CSC features. However, since major differences in the published xenotransplantation models do exist and the conclusion that CSCs are defined by their tumorigenic potential is still discussed controversially [19], this study focuses on the in vitro identification and characterization of stem cell-like cancer cells in established melanoma cell lines. We have addressed typical phenotypical and functional CSC features in established melanoma cell lines in order to identify cellular reagents amenable to detailed molecular profiling.

## Results

### Characterization of cell lines under investigation

The expression of melanoma associated antigens (MAAs) was used to characterize melanoma cell lines. The antigens of interest belonged to 2 main groups: tumor-associated cancer testis antigens (MAGE-A3, NY-ESO-1) and melanoma differentiation antigens (melanosomal matrix protein (gp100), melanoma antigen recognized by T cells (Melan-A/MART-1), tyrosinase)).D10, WM115, and HBL cell line expressed the melanoma differentiation antigens gp100, tyrosinase, and MART-1. These genes are also expressed in non-transformed melanocytes. In contrast, cancer testis antigens (CTA) are expressed in several malignancies of different histological origin and are also expressed on a few non-neoplastic cell populations including spermatogonia and trophoblasts, but not in healthy melanocytes. Results are shown in Table 1. The expression analysis of the regulatory core transcription factors NANOG, SOX2, and OCT4 revealed that a high NANOG expression was detectable in D10, WM115, and HBL cells. Results are shown in Table 2.

**Table 1:** Expression of MAA* in melanoma cell lines

**Cell line**	**Melanoma differentiation antigens**			**Cancer/testis antigens**	
	**gp100**	**tyrosinase**	**MART-1**	**MAGE-A3**	**NY-ESO**
**MZ2**	-	-	-	+ +	*-*
**D10**	+	+	+ +	+ +	*-*
**Me39**	-	-	+ +	+ +	*-*
**WM115**	-	-	+	+ +	*-*
**RE**	-	-	-	+ +	*-*
**Me59**	-	-	-	+/-	*-*
**Me67**	-	-	-	+ +	*-*
**Na8**	-	-	-	-	*-*
**HBL**	+	+	+ +	+ +	*-*

*MAA = melanoma-associated antigens; Expression levels were assessed using conventional PCR and evaluated semiquantitatively by densitometry. **gp100** = melanosomal matrix protein gp100; **tyrosinase** = key enzyme in melanin biosynthesis; **MART-1** = Melan-A/MART-1 = melanoma antigen recognized by T cells; **MAGE-A3** = melanoma antigen-A3 family, **NY-ESO** = cancer/testis antigen (see text); **(+)** = moderate expression; **(+ +)** = strong expression; **(-)** = no expression detectable.

**Table 2:** Expression of regulatory core transcription factors in melanoma cell lines

**Cell line**	**NANOG**	**OCT4**	**SOX2**
**MZ2**	-	-	-
**D10**	+ +	+	-
**Me39**	+ +	+ +	-
**WM115**	+	+	+
**RE**	+ +	+ +	-
**Me59**	-	+	-
**Me67**	+	-	-
**Na8**	-	-	-
**HBL**	+	-	-

**NANOG** = Nanog homeobox; **OCT4** = POU-domain transcription factor; **SOX2** = SRY (sex determining region Y)-box2; **(-)** = no expression (<10^-3^); **(+)** = moderate expression (≥10^-3^<10^-2^); **(+ +)** = strong expression (≥10^-2^). Expression level of TF gene reported as 2^–Ct^, where Ct = (Ct [TF gene] - Ct [ACTB]) sample.

### CD133 expression is detectable in metastatic melanoma cell lines

**Figure 1:**
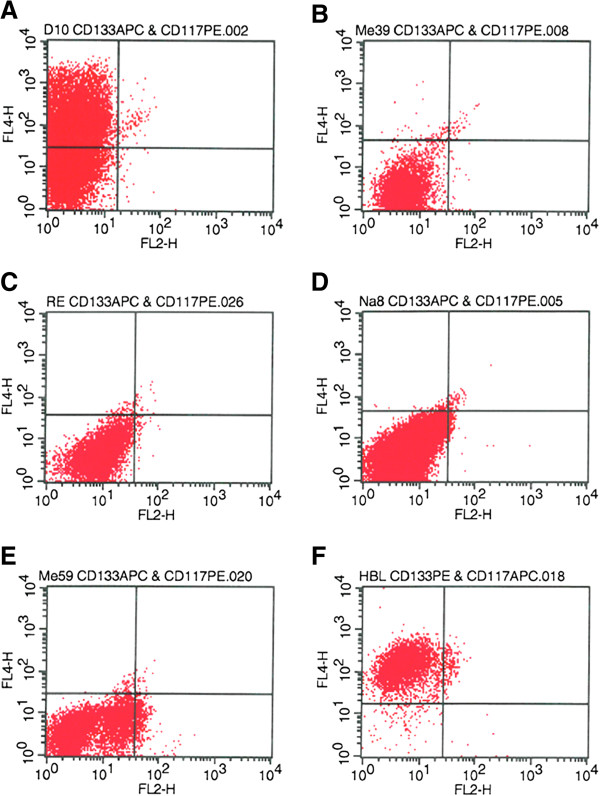
**Expression of CD133 and CD117 in melanoma cell lines.** Cell lines were stained with APC-and PE-conjugated monoclonal antibodies against the CD133 and CD117 epitope. The figure shows the dotplots of **A**: D10, **B**: Me39, **C**: RE, **D**: Na8, **E**: Me59 and **F**: HBL. Corresponding statistics are shown in Table 3.

Our data indicate that melanoma cell lines express discrete stem cell markers. However, the distribution of the expression was highly variable among the cell lines under investigation (Table 3). CD133 expressing could be detected in 5/9 melanoma cell lines including D10, Me39, RE, Me59, and Na8, decreasing from 80-1% of positive CD133+ subsets. Over 80% of D10 cells expressed CD133 (Figure 1A). In addition, nearly 2% of the Me39 cells (Figure 1B), more than 3% of RE cells (Figure 1C), and more than 1% of Na8 (Figure 1D) and Me59 (Figure 1E) cells also expressed CD133. Strikingly, CD105, the TGF-β receptor, was detectable on more than 75% of the cells of all melanoma cell lines under investigation with the exception of HBL. Na8 and HBL showed peculiar patterns of CD105, CD271, and CD117 expression. Virtually 83% of Na8 cells bound anti-CD271 antibody as opposed to 2% of HBL cells. In stark contrast, CD117 (c-kit) expression levels displayed an opposite pattern with less than 1% positivity in Na8 cells and more than 99% in HBL. In general, HBL emerged as an outstanding cell line in our panel since virtually all cells expressed CD117 with relatively high intensities (Figure 1F). No other surface marker under investigation was found to be expressed in these cells under these conditions. WM115 was previously reported to contain a CD133+ subpopulation [20]. This could not be confirmed in our experiments. Nonetheless, 2 subpopulations were identified in WM115. One fraction stained positive for CD105 (88%) and another one for CD271 (45%). The corresponding mean fluorescence intensities (MFI values) are attached as additional file (see Additional file 1).

**Table 3:** Phenotypical characterization of melanoma cell lines

**Cell line**	**CD133**	**CD105**	**CD146**	**CD271**	**CD117**
**MZ2**	neg.	86.41	73.92	99.74	neg.
**D10**	89.66	77.14	98.79	neg.	neg.
**Me39**	1.88	95.65	100.00	7.40	neg.
**WM115**	neg.	87.60	99.6	45.24	neg.
**RE**	3.11	74.84	98.31	59.96	1.28
**Me59**	1.00	75.55	36.40	2.72	18.95
**Me67**	neg.	75.94	73.76	10.75	neg.
**Na8**	1.05	97.22	0.67	82.54	neg.
**HBL**	neg.	22.66	30.08	2.32	99.46

Expression levels of fluorochrome-conjugated monoclonal antibodies against surface markers of interest on melanoma cell lines. Frequency and distribution of cells carrying surface markers expressed as percentages of positive gated cells; “neg.” indicates percentages of positive gated cells <1%.

**Figure 2:**
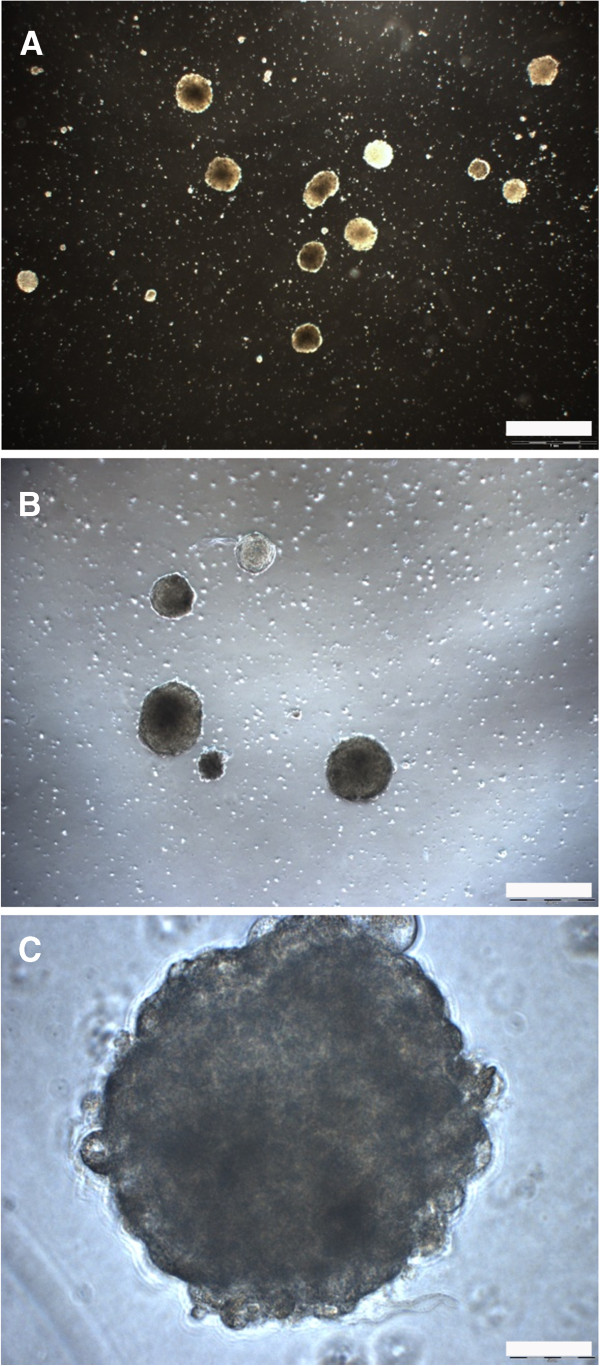
**Na8 spheroids in poly-HEMA-coated tissue culture flasks. A**: 2× magnification (scale bar 1 mm). **B**: 4× magnification (scale bar 500 μm). **C**: 40× magnification (scale bar 50 μm).

### D10, Na8 and HBL cells grow in spheroids

**Figure 3:**
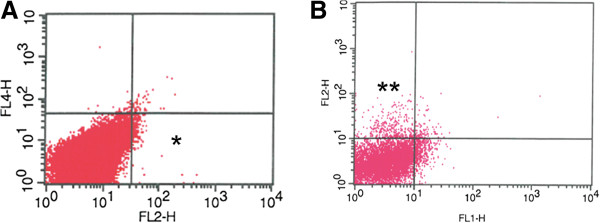
**CD133 expression in Na8 cells after 2D and 3D culture.** Dotplots of CD133-Expression (PE-conjugated) after **A**: monolayer culture, **B**: 3D culture (spheroidal growth). Following 3D- culture in pre-treated tissue culture flasks, almost 5% of the Na8 cells stained positive for CD133 compared to 1%.

Evidence of the capacity of CSCs to grow in spheroid structures has been repeatedly reported (refer to Introduction and Materials and Methods). To assess these features in the melanoma cell lines under investigation, cell lines were cultured in flasks pretreated with poly-HEMA, preventing attachment to the plastic surface. In these conditions, the D10 cell line - as well as the Na8 and HBL cell line - were clearly generating spheroids (Figure 2A-C). Furthermore, the phenotypes of these 3 cell lines were assessed upon 3D culture. Cells were labeled again with mAbs against CD133, CD117, CD105, CD271, and CD146; the results were compared with the staining observed in cells cultured in 2D (Table 3). Interestingly, the only modification observed in 3D as compared to the 2D cultures was represented by a modest increase of the fraction of CD133+ cells in the Na8 cell line (Figure 3). In contrast, expression levels of CD105, CD117, CD146, and CD271 were unmodified (data omitted for simplicity).

### CD133 expression in D10 cells correlates with clonogenicity

**Figure 4:**
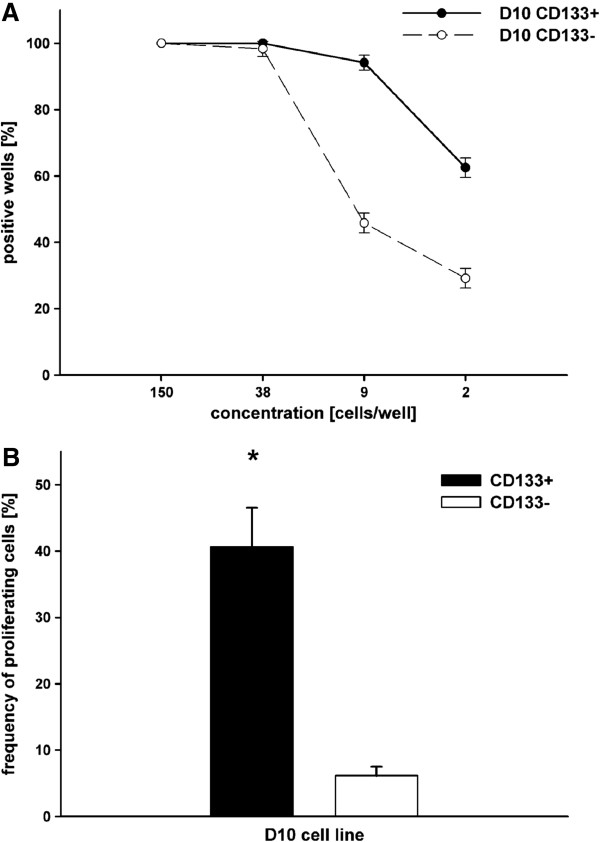
**Limiting dilution analysis and clonogenic assay. A**: Limiting dilution analysis. Percentage of positive wells (positive well = 1 cell colony/well) at different cell concentrations of CD133+ and CD133- D10 cells for calculating Poisson’s distribution (Figure 4B). **B**: Clonogenic capacity of D10 cells. Results of Poisson’s distribution with the frequency of proliferating cells in CD133+ (black column) and CD133- (white column) D10 cells, results expressed as mean percentages ± SD; (*) = p ≤ 0.001.

Clonogenic assays were performed by limiting dilution analysis (LDA) on cells magnetically sorted according to their expression of selected markers (Figure 4A). The frequency of proliferating cells was evaluated by Poisson’s distribution. Poisson’s distribution yielded that 40.60% ± 2.63 of the CD133+ cells were capable of giving rise to cell colonies, as opposed to 6.15% ± 0.598 of the negative fraction (Figure 4B). CD133+ D10 cells have a statistically significant higher clonogenic capacity as compared to CD133- D10 cells (p ≤ 0.001). Taken together, the expression of CD105 was not associated with a significantly higher clonogenic capacity in the cell lines investigated. In contrast, the CD105- fraction in Me39, WM115, Na8, and HBL cell lines even dominated in terms of clonogenic capacity, but not significantly. Except for the RE cell line where all CD271- cells were capable of forming colonies, CD271+ and CD271- cells possessed equal clonogenic potential. CD117 was expressed on almost all HBL cells. Notably, the absence of CD117 expression was not compatible with the in vitro survival of those cells. Referring to Poisson’s distribution, almost all HBL cells expressing CD117 are capable of inducing colony growth.

### CD133+ D10 cells induce tumor growth in vivo

**Figure 5:**
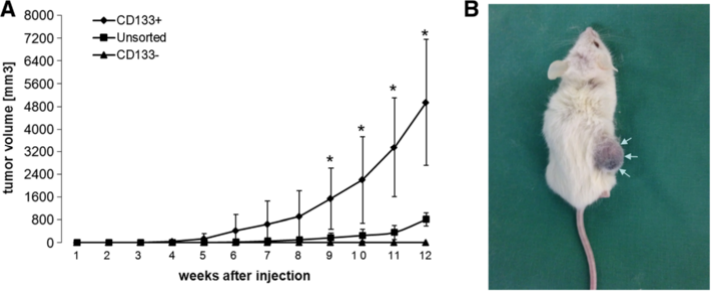
**Tumorigenicity of D10 cells. A**: The Change in tumor volume over a period of 12 weeks is shown in the graph. (*) = p ≤ 0.05. Bars represent SD. Tumor progression of CD133+ D10 cells significantly (p ≤ 0.05) differs from unsorted D10 tumors. **B**: Injection of CD133+ D10 cells into the right flank (R) leads to tumor formation (white arrows) while CD133- D10 cells are not capable of inducing tumor growth. Bilateral injection of unsorted D10 cells is followed by bilateral tumor formation.

**Figure 6:**
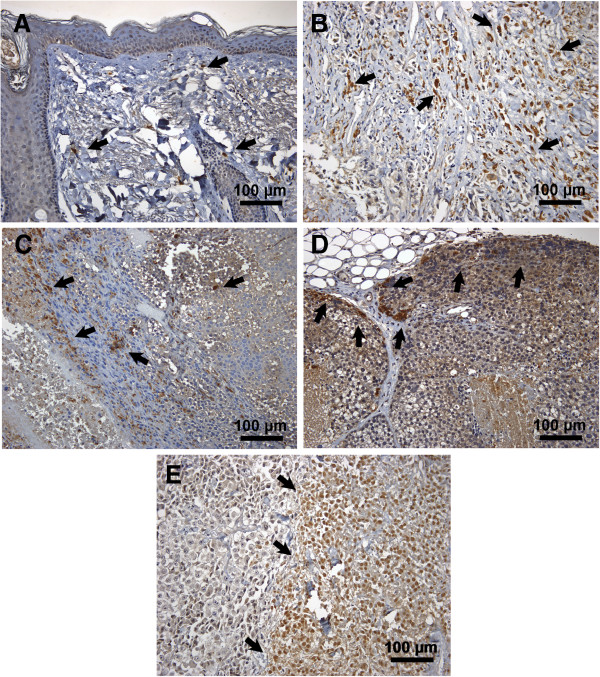
**Immunohistochemistry of xenografts and patient samples.** CD133 expression (brown spots, black arrows) in different tissue samples: **A**: normal skin section showing a few brown spots. **B**: primary melanoma tissue section, **C**: melanoma lymph node metastasis and **D**: xenograft induced by CD133+ D10 cells, all showing intense positivity for CD133. **E**: xenograft induced by unsorted D10 cells with less intense CD133+ staining. Scale bar 100 μm.

D10 cells were chosen from the panel of melanoma cell lines since they frequently expressed CD133 with a significantly higher clonogenic capacity and for their ability to grow in spheroids. In vivo tumor formation and growth could be observed with CD133+ D10 cells and unsorted D10 cells. CD133- D10 failed to induce tumor growth. Furthermore, isolated CD133+ D10 cells showed an accelerated growth compared to unsorted D10 cells (Figure 5). Xenografts induced by CD133+ D10 cells strongly stained positive for CD133 as shown by immunohistochemistry (Figure 6D). In contrast, CD133 expression in xenografts induced by unsorted D10 cells was less intense (Figure 6C). Additionally, CD133 expression was also detected in sections of a primary human melanoma (Figure 6B) and a lymph node metastasis (Figure 6E) but hardly in normal skin sections (Figure 6A).

### MGP expression is upregulated in CD133+ D10 cells

Following chip data processing and statistical analysis using Fisher’s ANOVA, 68 up- and 46 downregulated genes in CD133+ as compared to CD133- D10 cells (+/-1.3-fold, p ≤ 0.001) were obtained. The list of all differentially expressed genes is provided as additional file (Additional files 2 and 3). Two genes were outstandingly upregulated, i.e., matrix GIa protein (MGP) and prominin 1 (*PROM1*, CD133). The expression of MGP in CD133+ D10 cells and the fold of change of expression in relation to CD133- cells could be confirmed by PCR (see Additional file 4). A number of other genes upregulated in CD133+ D10 cells encode proteins involved in cell proliferation, including insulin-like growth factor-1 (IGF-1) and its binding protein insulin-like growth factor-binding protein-3 (IGFBP-3). Downregulated genes included those encoding tenascin C and TIMP1. Interestingly, the expression of *BCL2A1*, a gene that encodes a member of the pro- and antiapoptotic BCL-2 protein family, was downregulated.

### Categorization of differentially expressed genes

**Figure 7:**
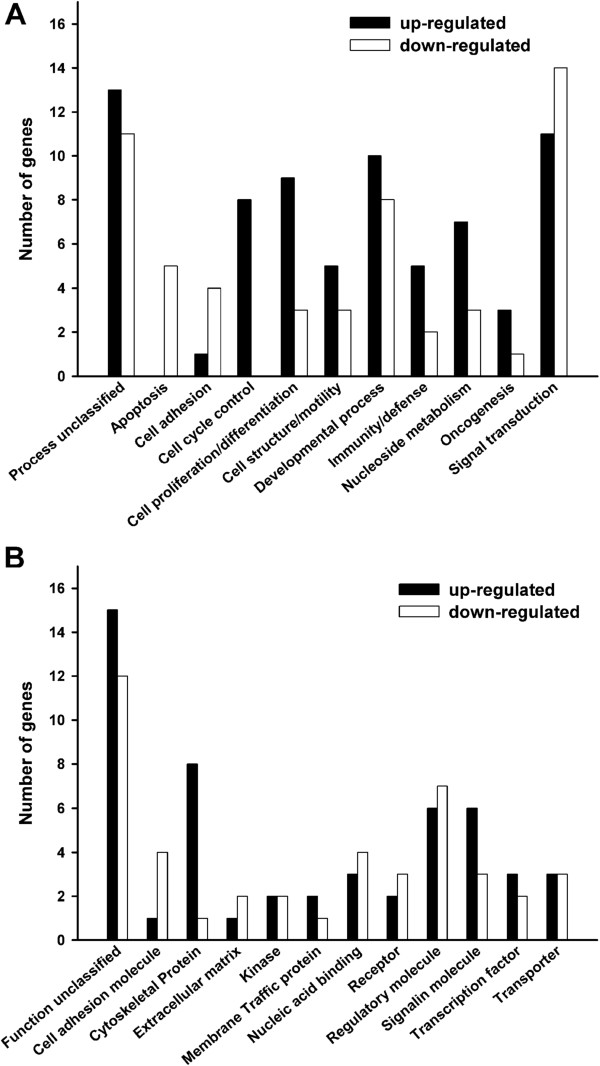
**Categorization of differentially expressed genes, detected in CD133+ D10 cells.** Number of genes encompassed with a specific **A**: molecular function and **B**: biological process. Black columns: upregulated genes. Gray columns: downregulated genes.

Differentially expressed genes were categorized by their molecular function (Figure 7A) and the biological processes (Figure 7B) that they are involved in by using the PANTHER classification system. Three of the 68 upregulated genes (*HJURP*, *LL22NC03-75B3.6*, and *DCC1*) and 2 of the 46 downregulated genes (*GOM1* and *CINP*) could not be identified by PANTHER. All differentially expressed genes and their symbols are provided as additional files.

## Discussion

This study aimed at investigating whether established melanoma cell lines contain tumor cell subsets that can be referred to as CSCs. Since CD133+ melanoma cells are rare in clinical samples and difficult to isolate from surgical specimens, the expression of stem cell surface markers, in particular CD133, was analyzed in 9 well-established human melanoma cell lines, each and every one originally derived from human metastatic malignant melanoma. The selection of melanoma cell lines reflects the heterogeneity of the original tumors and includes highly differentiated cell lines (D10, WM115, HBL) expressing the melanoma differentiation antigens gp100, tyrosinase, and MART-1, and undifferentiated cell lines. The melanoma cell line named WM115 was included in the study because of its previous characterization by Monzani’s group in 2007 [20] including a CD133+ phenotype and a strong tumorigenic potential [21]. For further characterization of our cell lines, the expression of the regulatory core transcription factors NANOG, SOX2, and OCT4 was analyzed. Those genes form a regulatory core essential for maintenance of the undifferentiated state of stem cells and the process of stem cell self-renewal in a complex regulatory network [22-24]. Interestingly, high NANOG expression was detectable on the rather differentiated cell lines D10, WM115, and HBL, suggesting that either these cell lines have been misclassified previously or the overexpression of NANOG might not be obviously linked to the state of differentiation of individual melanoma cell lines. Further studies are necessary to uncover the role of these transcription factors in melanoma cell lines.

Melanoma cell lines do express stem cell associated surface markers; however, their distribution was highly variable. Surprisingly, the expression of CD133 on WM115 cells was not detectable under the conditions used in this study. In contrast with the general thinking that CD133+ CSCs may represent only a minimal part of the total tumor cell population, CD133 was expressed on high percentages of D10 cells and very small percentages of Me39, RE, Me59, and Na8 cells. CD117 was expressed on virtually all HBL cells indicating that this might represent a specific feature of this highly differentiated cell line. Functional analysis of the surface markers used in this study revealed that only CD133+ D10 cells constantly demonstrated a significantly higher clonogenic capacity as compared to the CD133- fraction. The clonogenic capacity of the other markers (CD105, CD146, and CD117) throughout the cell lines was highly variable or oppositional in the samples examined. In a recent publication, CD271+ melanoma stem cells were found to be associated with metastasis, heterogeneity, and long-term growth [25]. In our panel, CD271+ cells could be identified in all cell lines except for D10. However, CD271+ cells did not demonstrate a significantly higher clonogenic capacity as compared to their negative counterparts.

Since the expression of CD133 was associated with a significantly higher clonogenic capacity in D10 cells the tumorigenic potential of this subset was investigated in vivo. CD133+ and unsorted D10 cells induced tumor formation in vivo. Shown by immunohistochemistry, xenografts induced by CD133+ D10 cells stained positive for CD133, confirming the conservation of this marker during tumor formation. The results of our study in which the tumorigenic potential of a CD133+ subset is demonstrated contrary to the CD133- fraction coincides with the classical cancer stem cell hypothesis and most articles published in this area. However, according to recent publications, the CD133- subset is also capable of conversely inducing tumor growth upon transplantation. Furthermore, during a suggested metastatic transition, originally CD133- induced tumors can transform to CD133+ xenografts. Two explanations of this phenomenon have been suggested so far: (1) the process of tumor initiation is a developmental process in which the CD133- subset gains tumorigenic capacity in the host, most likely through the influence of the adjacent environment or niche [26,27]. (2) CD133 expression does not identify the entire population of tumor-initiating cells [28]. In this context, future investigations on these particular CD133 subsets of D10 cells might help to both, uncover the role of the tumor niche during tumorigenesis and to help to explain the phenomenon of marker transformation in vivo.

Based on these results, gene expression profiling using gene chips was performed on CD133+ and CD133- D10 cells. MGP (32.4-fold) and positive control PROM1 (CD133; 27.7-fold), were outstandingly upregulated across the other 68 upregulated genes. In addition, IGF-1 and its major corresponding binding protein IGFBP-3 were, respectively, 3.5 and 2.7-fold upregulated in CD133+ D10 cells as compared to the CD133- fraction. IGF-1 plays a key role in the development and growth of multiple tumors and in the prevention of apoptosis. In melanoma cells, IGF-1 has been shown to mediate resistance to anoikis, a form of programmed cell death, which is induced by anchorage-dependent cells detaching from the surrounding extracellular matrix. Recently, Hilmi and co-workers [29] also demonstrated that IGF-1 promotes resistance to apoptosis in melanoma cells through an increased expression of BCL2, BCL-X (L), and survivin. Inconsistently with findings published by Fang’s group [14], CD133+ D10 cells had not upregulated the expression of ABCG2 (ATP-binding cassette, sub-family G (WHITE), member 2A), which was identified to be overexpressed in primary or metastatic melanoma compared to benign melanocytic nevi [18].

## Conclusions

Taken together, our data suggest that established melanoma cell lines represent useful tools for the investigation of functional features of CSCs. In particular, the CD133+ subset of D10 cell line with a significantly higher clonogenic and tumorigenic capacity might qualify as melanoma cancer stem cell model. However, since the CD133- subset failed to induce xenografts the role of the tumor niche for this particular subset needs to be evaluated in future studies. Furthermore, gene profiling of the CD133+ subset of the D10 melanoma cell line has resulted in the identification of 1 gene, i.e., *MGP*, consistently upregulated, in comparison with the CD133- subset of the same cell line. Further in vitro and in vivo studies are warranted to validate these results at the gene and protein level and to assess the potential diagnostic and prognostic relevance of *MGP*, CD133, and IGF expression in clinical melanoma specimens.

## Methods

### Cell culture

A panel of melanoma cell lines representative of tumors at diverse differentiation stages was selected. All cell lines were derived originally from metastatic melanomas. The WM115 cell line was obtained from the ATCC; MZ2 cell line was a gift from Dr. van der Bruggen (Brussels, Belgium), whereas HBL, Na8, and D10 were provided by Dr. Eberle (Basel, Switzerland). RE, Me39, Me59, and Me67 cell lines were generated by Giulio Spagnoli’s group (Basel, Switzerland). Cell lines were cultured in Gibco® DMEM (Invitrogen AG, Basel, Switzerland; containing 4.5 g/L glucose and NEAA), supplemented with 10% FBS (Invitrogen AG, Basel, Switzerland), 1% sodium pyruvate (Invitrogen AG, Basel, Switzerland), 1% HEPES buffer (Invitrogen AG, Basel, Switzerland), and 2% PSG (Invitrogen AG, Basel, Switzerland). For investigating 3-dimensional (3D) spheroidal growth, tissue culture flasks (75 cm^2^, tissue culture flasks) (TPP, MIDSCI, St. Louis, MO, USA) were pretreated with poly-HEMA (Sigma-Aldrich®, St. Louis, MO, USA) according to the manufacturer’s instructions. Cell lines were also cultured in both 20% and 1% oxygen humidified atmosphere at 37°C.

### Fluorescent-activated cell sorting (FACS)

Cell suspensions from melanoma cell lines were incubated with the following fluorochrome-conjugated antibodies: PE/APC-CD133, FITC-CD105, APC-CD271, FITC-CD146, and APC-CD117 (Becton-Dickinson, San José, CA, USA). Between 5 × 10^5^ and 5 × 10^6^ cells per tube were labeled with 5 μL of labeled monoclonal antibodies (mAbs) of interest. Cells were fixed by in 1% paraformaldehyde (PAF). The phenotype was assessed using a FACSCalibur® flow cytometer (Becton-Dickinson).

### Limiting dilution and clonogenic assay

In order to assess the possible differences in the clonogenic capacity of cells carrying selected surface markers, melanoma cell lines were incubated with MicroBeads® loaded with CD105, CD133, CD271, and CD117 (Miltenyi Biotec, Bergisch Gladbach, Germany) and applied to columns allowing their magnetic separation into positively and negatively labeled fractions by using a MiniMACS™ separation unit (Miltenyi Biotec, Bergisch Gladbach, Germany) according to established protocols. In this study, cell suspensions of melanoma cell lines were diluted serially. Cell counts were carried out after 14 days. In this study, positive results (positive wells) were determined as at least 1 cell colony per well. The frequency of proliferating cells for each target phenotype was assessed by applying Poisson’s distribution [30]. Frequency of proliferating cells was expressed as mean ± SD. Differences between groups were assessed by one-way analysis of variance (ANOVA), and differences within each group were analyzed by one-way repeated-measures ANOVA. To isolate overall differences, appropriate differences were considered significant at p ≤ 0.001.

### Animal experiments and immunohistochemistry

All animal experiments were carried out under anaesthesia by intraperitoneal injections of 0.1 mL saline solution (Sigma, Steinheim, Germany) per 10 g body weight containing 90 mg/kg body weight ketamine hydrochloride (KetavetVR ; Parke Davis, Freiburg, Germany) and 25 mg/kg body weight dihydroxylidinothiazine hydrochloride (RompunVR; Bayer, Leverkusen, Germany). Conduction of the experiments was approved by the institutional ethical committee and the Federal Office for Consumer Protection and Food Safety with the reference number 33.9-42502-04-11/0401. Magnetically sorted 1× 10^6^ CD133+ D10 cells (MicroBeads®, Miltenyi Biotec, Bergisch Gladbach, Germany) were subcutaneously injected into the right flank regions of female NOD scid gamma mice (NSG; NOD.Cg-Prkdcscid Il2rgtm1Wjl/SzJ) and 1×10^6^ CD133- D10 cells in the contralateral region. Unsorted D10 cells (1×10^6^ cells respectively) were bilaterally injected as control group. Eight mice were assigned to each group. Tumor growth was assessed once a week using a caliper and the actual tumor mass was estimated by calculating the volume according to the ellipsoid volume formula: 4/3 π (½ × lenght × width × height). Statistical analysis was carried out using ANOVA. Mice were euthanized after 12 weeks or in case of fulminate tumor growth. Xenografts were harvested for immunohistochemistry. For detection of CD133 formalin-fixed specimens were embedded in paraffin and cut into five-μm-thick sections. The sections were incubated with a rabbit anti-human PROM1/CD133 antibody (Abnova, Taipei, Taiwan). A biotin-conjugated goat anti-rabbit antibody (Dianova, Hamburg, Germany) was used as secondary antibody. Incubation with streptavidin–horseradish peroxidase (Dianova) was followed by color development with 3, 3’-diaminobenzidine (DAB) substrate (Vector Laboratories, Burlingame, USA) at room temperature. The sections were counterstained with hemalaun (Merck KGaA, Darmstadt, Germany) and examined by light microscopy (DM4000B, Leica, Wetzlar, Germany). For negative control the primary antibody was omitted. All control stainings were negative.

### Gene expression

The expression of the genes encoding the melanoma-associated antigens (MAAs) gp100, tyrosinase, and Melan-A/MART-1 and the cancer/testis antigens (CTA) MAGE-A3 and NY-ESO was assessed by conventional PCR, its extent was semiquantitatively evaluated by densitometry as previously described by Spagnoli [31,32]. Primers and probes for the housekeeping gene ß-actin (hACTB) as well as for NANOG, OCT4, SOX2, and MAGE-3 and MGP were provided by Assays-on-Demand, Gene Expression Products (Applied Biosystems, Foster City, CA, USA). The other primer sequences (gp100, tyrosinase, Melan-A/MART-1, and NY-ESO) were derived from existing literature, as indicated below or were generated using appropriate software (Primer Express™, Applied Biosystems, Foster City, CA). Sequences of primers and probes and Assays-on-Demand are attached as additional files (see Additional files 5 and 6). TaqMan® analysis was carried out on a 7900HT Sequence Detection System. Singleplex PCR reactions were performed in Fast Gene Quantification in 96-Well Plates (the thermal cycling conditions included a step of 20 s at 95°C followed by 40 cycles of 95°C for 1 s and 60°C for 20 s) with The TaqMan® Fast Universal PCR Master Mix (10 μl) in a volume of 20 μl containing 2 μl of cDNA and 1 μl of specific TaqMan® Gene Expression Assay. All reactions were performed in triplicate. All reagents were from Applied Biosystems (Foster City, CA, USA). The comparative Ct method by Pfaffl [33] was employed to determine the MGP expression in CD133+ and CD133- D10 cells. The MGP expression in CD133- D10 cells was identified as a calibrator sample and the CD133+ sample expressed n-fold mRNA relative to the calibrator. Final amounts of target were determined as follows: target amount = 2^–Ct^, where C_t_ = [C_t_ (MGP D10 CD133+) – C_t_ (ACTB)]_sample_ – [C_t_ (MGP D10 CD133-) – C_t_ (ACTB)]_calibrator_.

### Cell sorting for microarray studies

CD133+ and CD133- D10 cells were sorted using a FACSVantage® Cell Sorter for genome-wide gene expression profiling. About 40 × 10^6^ D10 cells were isolated and resuspended in 25 μL PBS containing 2% FBS for 1 × 10^6^ cells. Afterwards, D10 cells were labeled with 2.5 μL fluorochrome-linked mAbs against CD133 (CD133/2PE; Miltenyi Biotech, Bergisch Gladbach, Germany) for 1 × 10^6^ cells. D10 cells were resuspended in 5 mL polyethylene tubes (BD Falcon™, BD Biosciences, San Jose, CA, USA) at a concentration of 10^7^ cells/mL in a sorting solution. To avoid cells from sticking to the tube’s inner surface, 5 mL polyethylene tubes were coated with 1% BSA; in order to maintain single cell suspension and prevent cell clumping, the sorting solution based on PBS, contained 0.5% BSA and 5 mM EDTA. CD133+ and CD133- D10 cells were sorted out in duplicate by the FACSVantage® cell sorter until 10^6^ cells of each condition were collected in the DMEM in uncoated polyethylene 5 mL tubes (BD, Falcon). The results of the cell sorting are attached as additional file (see Additional file 7).

### Microarray studies

Genome-wide gene expression profiling was carried out according to MIAME-standards (Minimal Information about an Array Experiment) using Affymetrix GeneChips® Human Genome U133A 2.0 expression arrays (Affymetrix, UK Ltd.). The experimental design and related protocols are published in a MIAME-compliant format and can be reviewed on ArrayExpress® (Accession Number: E-MEXP-3542). Briefly, total RNA was isolated from previously sorted CD133+ and CD133- D10 cells by using an RNeasy Mini Kit (Qiagen, Basel, Switzerland) following manufacturer’s protocols. After elution, a total RNA cleanup was performed using an RNeasy®MinElute® Cleanup Kit (Cat # 74204). The RNA yield was quantified by spectrophotometric analysis (NanoDrop® Technologies, Wilmington, USA) using the convention that 1 absorbance unit at 260 nm equals to 40 μg/mL RNA. Aliquots of each RNA sample were saved for RNA fragment analysis in an Agilent Bioanalyzer 2100® (Agilent Technologies, Basel, Switzerland) by using an RNA 6000 Nano Kit (Agilent Technologies, Basel, Switzerland).

### Target synthesis

Biotin-labeling of RNA was performed as described in the GeneChip® Expression Analysis Technical Manual (Affymetrix, Santa Clara, USA). Double-stranded cDNA was synthesized according to the One-Cycle cDNA Synthesis Kit (Affymetrix, Cat # 900431), starting from 5 μg of the total RNA. The material was purified with a Sample Cleanup Module (Affymetrix, Cat # 900371). Purified cDNA was used for an in vitro transcription reaction by using an IVT labeling kit (Affymetrix, Cat # 900449). The hybridization cocktail (130 μL) containing fragmented biotin-labeled target cRNA at a final concentration of 0.05 μg/μL was transferred into an Affymetrix Human Genome U133A 2.0 and incubated at 45°C on a rotator in a hybridization oven 640 (Affymetrix) for 16 h at 60 rpm. The arrays were washed and stained on a Fluidics Station 450 (Affymetrix) by using a Hybridization Wash and Stain Kit (Affymetrix, Cat # 900720). In order to increase the signal strength, the antibody amplification protocol (FS450_0002) was used.

### Chip data processing

Gene chips were processed with an Affymetrix GeneChip® Scanner 3000 7G (Affymetrix) and DAT image files of the microarrays were generated using GeneChip® Operating Software (GCOS 1.4; Affymetrix). Within GeneSpring® raw data were preprocessed including background adjustment, normalization, and summarization of probe sets, using the GeneChip® Robust Multiarray Analysis (GC-RMA). Genes whose signals were lower than background in all gene chips were filtered out, subsequently genes were filtered based on fold change. Statistical analysis on the gene expression profile were performed by using Fisher’s analysis of variance (ANOVA). Profiles of genes significantly (p ≤ 0.001) up- or downregulated (+/-1.3-fold) in CD133+ D10 cells as compared to CD133- D10 cells were obtained. In addition, final gene expression data were analyzed using the Protein Analysis Through Evolutionary Relationships (PANTHER) classification system [34].

## Abbreviations

ABCG2: ATP-binding cassette, sub-family G (WHITE), member 2A; ABCA: ATP-binding cassette, sub-family A (ABC1); ANOVA: analysis of variance; BSA: Bovine serum albumin; CSC: Cancer stem cell; CD: Cluster of differentiation; CTA: Cancer/testis antigen; CTLA-4: Cytotoxic T-lymphocyte antigen-4 (CD152); DMEM: Dulbecco’s modified Eagle Medium; DNA: Deoxyribonucleic acid; EDTA: Ethylene diamine tetra acetic acid; FACS: Fluorescent-activated cell sorting; gp100: Melanosomal matrix protein; HEPES: 4-(2-hydroxyethyl)-1-piperazineethanesulfonic acid; IGF-1: Insulin-like growth factor-1; IGFBP-3: Insulin-like growth factor-binding protein-3; IL-2: Interleukin-2; MAA: Melanoma-associated antigens; (mAbs): Monoclonal antibodies; MAGE-3: Melanoma-associated antigen-3; Melan-A/MART-1: Melanoma antigen recognized by T-cells; MFI: Mean fluorescence intensity; MGP: Matrix GIa protein; mM: Millimolar; NANOG: North american network operators’ group (transcription factor); NEAA: Non-essential amino acid; NY-ESO: New York esophageal; OCT4: Octamer-binding transcription factor 4; RT-PCR: Reverse transcriptase-polymerase chain reaction; poly-HEMA: Poly (2-hydroxyethyl methacrylate); PFA: Paraformaldehyde; PANTHER: Protein analysis through evolutionary relationships; PBS: Phosphate-buffered saline; PROM1: Prominin 1 (CD133); PSG: Penicillin, streptomycin, and gentamycin; SOX2: SRY (sex determining region Y)-box 2 (transcription factor); TGF-β: Transforming growth factor beta.

## Competing interests

The authors declare that they have no competing interests.

## Authors’ contributions

RMZ conducted the in vitro experiments and wrote the paper. PK carried out the PCR and conducted the animal experiments. PD performed the microarrays. AK took care of the immunohistochemistry. HK carried out the statistical analysis and the literature review. AE carried out animal surgeries. NCG and FT supervised the project and edited the manuscript. All authors read and approved the final manuscript.

## Supplementary Material

Additional file 1 **Phenotypical characterization of melanoma cell lines. Corresponding mean fluorescent intensities (MFI).** MFI = signal (mAb) - signal (isotype control). No signal detected was indicated by (neg.) when MFI < 1. Click here for file

Additional file 2 **Results of gene expression profiling.** Table shows significantly (p ≤ 0.001) upregulated genes (>1.3-fold) compared to CD133- D10 cells. FC = fold change of up-regulation; RefSeq = References to multiple sequences; ChrLoc = chromosomal location. The gene CEACAM1 is provided as a triplet, the genes ENTPD and TPD52 as duplicates, respectively. Click here for file

Additional file 3 **Results of gene expression profiling.** Table shows significantyl (p ≤ 0.001) down-regulated genes (>1.3-fold) compared to CD133- D10 cells. FC = fold change of up-regulation; RefSeq = References to multiple sequences; ChrLoc = chromosomal location. Click here for file

Additional file 4 **Real-time rt-PCR results for MGP expression in CD133+ (black column) and CD 133- (white column) D10 cells.** (*) = p ≤ 0.05. Click here for file

Additional file 5 **Primers and Probes.** Gp100 = melanosomal matrix protein gp100; MART-1 = Melan-A/MART-1 = melanoma antigen recognized by T cells; tyrosinase = key enzyme in melanin biosynthesis; NY-ESO = cancer/testis antigen (see text). Click here for file

Additional file 6 Gene expression assays* for real-time RT-PCR. *TaqMan® Gene Expression Assays (Assay-on-demand®; see Assay IDs), Applied Biosystems ([AB], Foster City, CA); hACTB = human ACTB (beta actin) endogenous control; NANOG = nanog homeobox; OCT4 = POU-domain transcription factor; SOX2 = SRY (sex determining region Y)-box 2; MAGE-A3 = melanoma antigen-A3 family; MGP = Matrix GIa protein. Click here for file

Additional file 7 **Results of FACSVantage® cell sorting of CD133+ and CD133- D10 cells.** Fluorochrome-linked mAbs against CD133 (CD133/2PE) were used. **A:** Histogram **B:** dotplots. Click here for file
