# Supplementary material for: Functional features of cancer stem cells in melanoma cell lines
Source: Cancer Cell Int. 2013 Aug 6;13:78. doi: 10.1186/1475-2867-13-78 (PMC3765139; doi:10.1186/1475-2867-13-78)
Supplement: Additional file 1 — Phenotypical characterization of melanoma cell lines. Corresponding mean fluorescent intensities (MFI). MFI = signal (mAb) - signal (isotype control). No signal detected was indicated by (neg.) when MFI < 1. [file 1475-2867-13-78-S1.doc]

**Additional file:** Corresponding mean fluorescent intensities

| **Cell line** | **CD133** | **CD105** | **CD146** | **CD271** | **CD117** |
| --- | --- | --- | --- | --- | --- |
| **MZ2** | neg. | 16.66 | 14.01 | 499.9 | neg. |
| **D10** | 60.14 | 11.1 | 342.72 | neg. | neg. |
| **Me39** | neg. | 15.36 | 206.38 | 6.47 | neg. |
| **WM115** | neg. | 15.53 | 355.32 | 12.53 | neg. |
| **RE** | neg. | 12.45 | 686.77 | 14.11 | neg. |
| **Me59** | neg. | 15.69 | 7.38 | neg. | 3.95 |
| **Me67** | neg. | 11.21 | 16.04 | 1.25 | neg. |
| **Na8** | neg. | 32.81 | neg. | 51.38 | neg. |
| **HBL** | neg. | 4.46 | 5.75 | neg. | 81.83 |
| Single staining. Corresponding intensities of surface marker expression on melanoma cell lines (**Table 5a**). Results displayed as mean fluorescence intensity values (MFI): **MFI** = signal (mAb) - signal (isotype control). No signal detected was indicated by (neg.) when MFI < 1. | | | | | |
