# Supplementary figures and images for: Functional features of cancer stem cells in melanoma cell lines
Source: Cancer Cell Int. 2013 Aug 6;13:78. doi: 10.1186/1475-2867-13-78 (PMC3765139; doi:10.1186/1475-2867-13-78)

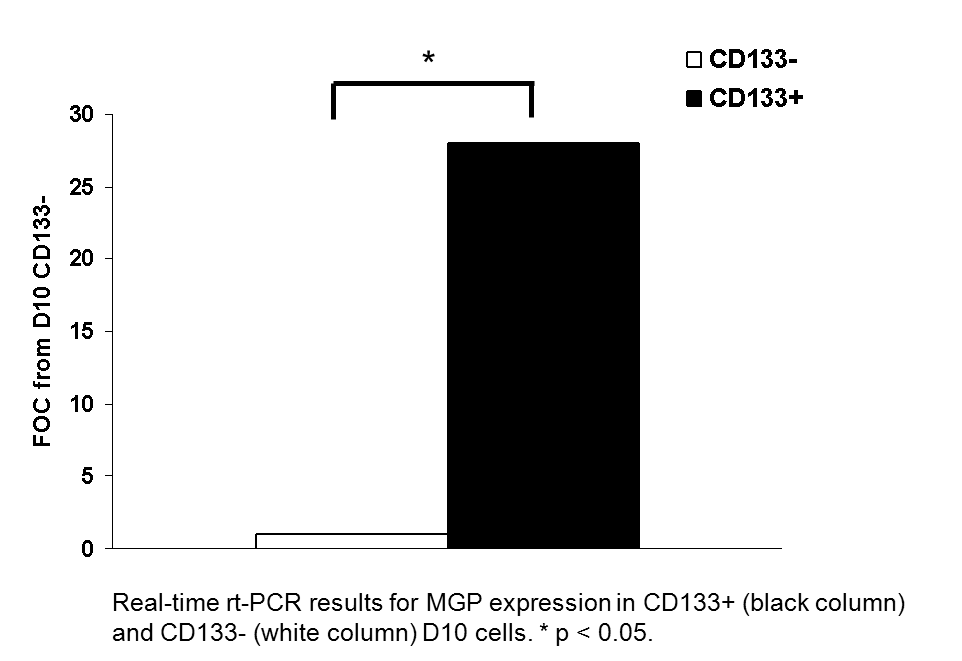

Supplement: Additional file 4 — Real-time rt-PCR results for MGP expression in CD133+ (black column) and CD 133- (white column) D10 cells. (*) = p ≤ 0.05. [file 1475-2867-13-78-S4.tiff]

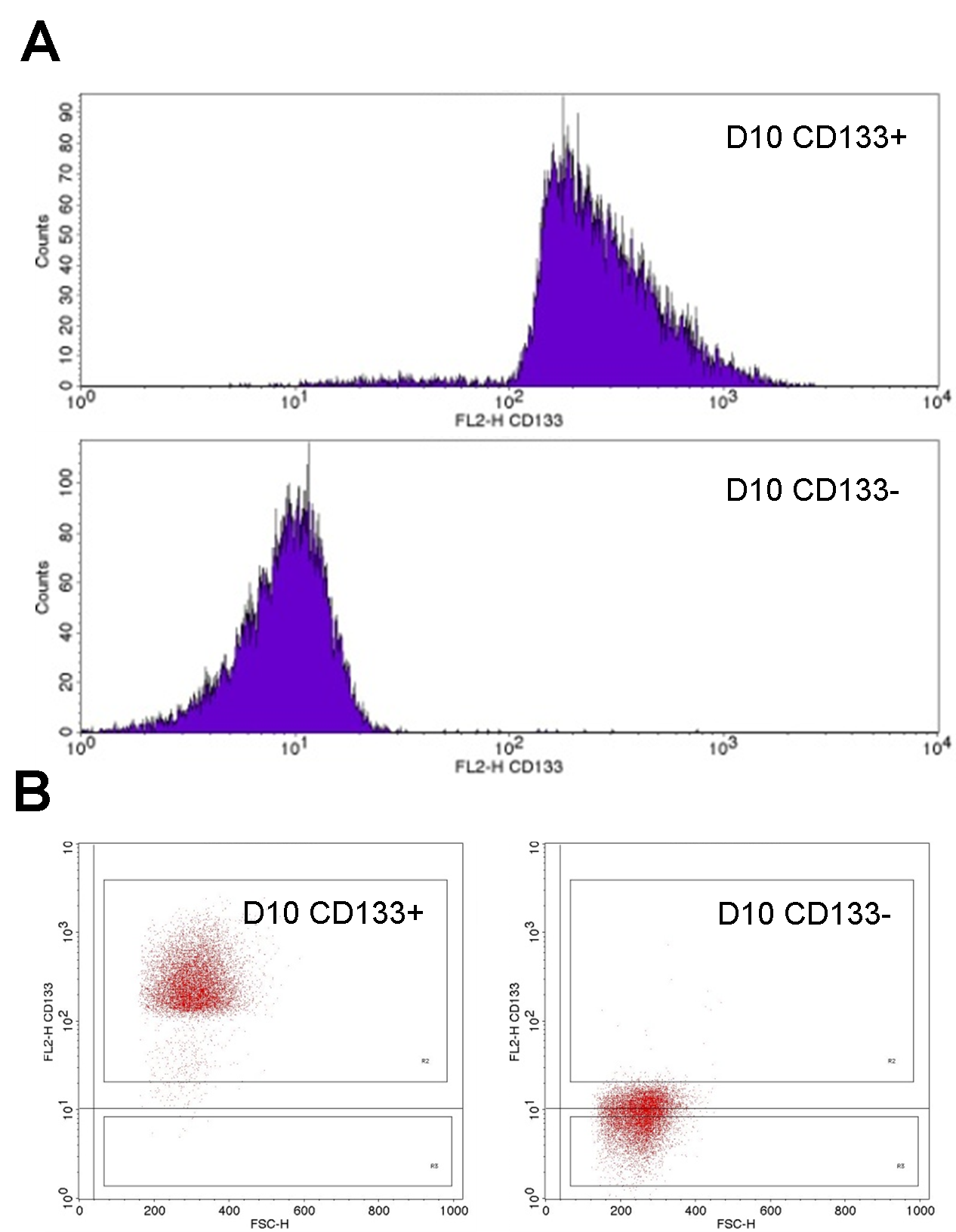

Supplement: Additional file 7 — Results of FACSVantage® cell sorting of CD133+ and CD133- D10 cells. Fluorochrome-linked mAbs against CD133 (CD133/2PE) were used. A: Histogram B: dotplots. [file 1475-2867-13-78-S7.tiff]
