# Supplementary material for: Functional features of cancer stem cells in melanoma cell lines
Source: Cancer Cell Int. 2013 Aug 6;13:78. doi: 10.1186/1475-2867-13-78 (PMC3765139; doi:10.1186/1475-2867-13-78)
Supplement: Additional file 5 — Primers and Probes. Gp100 = melanosomal matrix protein gp100; MART-1 = Melan-A/MART-1 = melanoma antigen recognized by T cells; tyrosinase = key enzyme in melanin biosynthesis; NY-ESO = cancer/testis antigen (see text). [file 1475-2867-13-78-S5.doc]

| **Gene** | **Forward and reverse primers (5`🡪3`)** | **Probe (5`🡪3`)** |
| --- | --- | --- |
| **18S rRNA** | CGGCTACCACATCCAAGGAAGCTGGAATTACCGCGGCT | TGCTGGCACCAGACTTGCCCTC |
| **gp100** | TCCCCCTGGATTGTCTTCTGCTCAAATGCATCCCCCTCA | CCCTGGACATTGTCCAGGGTATTGAAAGTGA |
| **MART-1** | TCTATGGTTACCCCAAGAAGGGGATCACTGTCAGGATGCCGA | ACGGCTGAAGAGGCCGCTGGGAT |
| **tyrosinase** | TTTGCCTGAGTTTGACCCAATAGAGGCATCCGCTATCCCA | TAGAAATACACTGGAAGGATTTGCTAGTCCACTTACTA |
| **NY-ESO** | GCTGAATGGATGCTGCAGACTGGAGACAGGAGCTGATGGA | TGTGTCCGGCAACATACTGACTATCCGA |
| **gp100** = melanosomal matrix protein gp100; **MART-1** = Melan-A/MART-1 = melanoma antigen recognized by T cells; **tyrosinase** = key enzyme in melanin biosynthesis**; NY-ESO** = cancer/testis antigen (see text). | | |
