# Supplementary material for: Functional features of cancer stem cells in melanoma cell lines
Source: Cancer Cell Int. 2013 Aug 6;13:78. doi: 10.1186/1475-2867-13-78 (PMC3765139; doi:10.1186/1475-2867-13-78)
Supplement: Additional file 6 — Gene expression assays* for real-time RT-PCR. *TaqMan® Gene Expression Assays (Assay-on-demand®; see Assay IDs), Applied Biosystems ([AB], Foster City, CA); hACTB = human ACTB (beta actin) endogenous control; NANOG = nanog homeobox; OCT4 = POU-domain transcription factor; SOX2 = SRY (sex determining region Y)-box 2; MAGE-A3 = melanoma antigen-A3 family; MGP = Matrix GIa protein. [file 1475-2867-13-78-S6.doc]

**Additional file:** Gene expression assays* for real-time RT-PCR

| **Gene symbol** | **Assay ID** |
| --- | --- |
| **hACTB** | Hs99999903_m1 |
| **NANOG** | Hs02387400_g1 |
| **OCT4** | Hs00742896_s1 |
| **SOX2** | Hs00602736_s1 |
| **MAGE-A3** | Hs00366532_m1 |
| **MGP** | Hs00969490_m1 |
| *TaqMan® Gene Expression Assays (Assay-on-demand®; see Assay IDs), Applied Biosystems ([AB], Foster City, CA); **hACTB** = human ACTB (beta actin) endogenous control; **NANOG** = nanog homeobox; **OCT4** = POU-domain transcription factor; **SOX2** = SRY (sex determining region Y)-box 2; **MAGE-A3** = melanoma antigen-A3 family; MGP = Matrix GIa protein. | |
